# Tumor Necrosis Factor-Related Apoptosis-Inducing Ligand-Induced Apoptosis in Prostate Cancer Cells after Treatment with Xanthohumol—A Natural Compound Present in *Humulus lupulus* L.

**DOI:** 10.3390/ijms17060837

**Published:** 2016-06-22

**Authors:** Małgorzata Kłósek, Anna Mertas, Wojciech Król, Dagmara Jaworska, Jan Szymszal, Ewelina Szliszka

**Affiliations:** 1 Chair and Department of Microbiology and Immunology, School of Medicine with Division of Dentistry in Zabrze, Medical University of Silesia in Katowice, Jordana 19, 41-808 Zabrze, Poland; mklosek@sum.edu.pl (M.K.); amertas@sum.edu.pl (A.M.); wkrol@sum.edu.pl (W.K.); djaworska@sum.edu.pl (D.J.); 2 Department of Production Engineering, Faculty of Materials Engineering and Metallurgy, Silesian University of Technology, Krasińskiego 8b, 40-019 Katowice, Poland; jan.szymszal@polsl.pl

**Keywords:** TRAIL, apoptosis, xanthohumol, prostate cancer cells

## Abstract

TRAIL (tumor necrosis factor-related apoptosis-inducing ligand) is an endogenous ligand, which plays role in immune surveillance and anti-tumor immunity. It has ability to selectively kill tumor cells showing no toxicity to normal cells. We tested the apoptotic and cytotoxic activities of xanthohumol, a prenylated chalcone found in *Humulus lupulus* on androgen-sensitive human prostate adenocarcinoma cells (LNCaP) in combination with TRAIL. Cytotoxicity was measured by 3-(4,5-dimethylthiazol-2-yl)-2,5-diphenyltetrazolium bromide tetrazolium reduction assay (MTT) and lactate dehydrogenase assay (LDH). The expression of death receptors (DR4/TRAIL-R1 and DR5/TRAIL-R2) and apoptosis were detected using flow cytometry. We examined mitochondrial membrane potential (ΔΨm) by DePsipher reagent using fluorescence microscopy. The intracellular expression of proteins was evaluated by Western blotting. Our study showed that xanthohumol enhanced cytotoxic and apoptotic effects of TRAIL. The tested compounds activated caspases-3, -8, -9, Bid, and increased the expression of Bax. They also decreased expression of Bcl-xL and decreased mitochondrial membrane potential, while the expression of death receptors was not changed. The findings suggest that xanthohumol is a compound of potential use in chemoprevention of prostate cancer due to its sensitization of cancer cells to TRAIL-mediated apoptosis.

## 1. Introduction

TRAIL (tumor necrosis factor-related apoptosis-inducing ligand) is a type II transmembrane protein identified by S. Wiley *et al.* in 1995 [1]. A few months later Pitti *et al.* [2] documented the same protein *in vivo* and named it Apo-2. TRAIL (Apo2L/TRAIL) in a native form is associated with the membrane cell and can be cleaved by metalloproteases (MMPs) to yield a soluble form [3].

So far, TRAIL expression has been detected in monocytes, macrophages, dendritic cells, natural killer (NK) cells, and activated T cells [4,5,6,7]. This ligand is characterized by the ability to induce apoptosis in tumor cells but shows no toxic effects to non-cancer cells [8,9]. TRAIL is involved in immune surveillance and anti-tumor immunity. TRAIL mRNA is expressed in the thymus, spleen, prostate, ovary, lungs, and colon, but not in the brain [10].

TRAIL in a homotrimer form can mediate apoptotic effects by binding to its receptors. Four transmembrane receptors and one soluble TRAIL receptor have been discovered to date, namely, TRAIL-R1, TRAIL-R2, TRAIL-R3, TRAIL-R4, and osteoprotegerin (OPG, TNFRSF11B) [11,12,13,14]. TRAIL-R1 and TRAIL-R2 are called “death receptors” because these receptors undergo trimerization binding TRAIL and initiate TRAIL-induced apoptosis. Other types of receptors are called “decoy receptors”. They bind TRAIL, but do not transmit a signal to apoptosis [15].

Numerous tumor cells are resistant to apoptosis mediated by TRAIL. The cause may be a low expression of “death receptors” or overexpression of “decoy receptor” [16,17,18]. In many cancer cells which are not sensitive to apoptosis mediated by TRAIL there are no correlations between expression of death or decoy receptors [19]. Other mechanisms of TRAIL-resistance include the overexpression of anti-apoptotic proteins, such as Bcl-2 or Bcl-xL which block the pro-apoptotic proteins by forming heterodimers with them. Anti-apoptotic Bcl-2 in this way prevents the increase of mitochondrial membrane permeability. On the other hand, deficiency of Bax or Bak expression evokes TRAIL-resistance in tumor cells [20,21,22,23,24]. Caspase activity also affects the sensibility of cancer cells to TRAIL-induced apoptosis [25].

Prostate cancer belongs to most commonly-diagnosed types of malignant tumors in men [26]. One of the causes of prostate cancer is probably the deregulation process leading to apoptosis. The aim of the prevention in patients with prostate cancer is induction of apoptosis in cancer cells [27,28]. Prostate cancer cells are a recognized model for studying *in vitro* the effect of overcoming resistance to TRAIL ligand.

The term “chemoprevention” was introduced by Sporn in 1976 [29]. He postulated that natural dietary compounds and/or synthetic pharmacological agents can arrest or reverse the process of carcinogenesis. Flavonoids, secondary metabolic products in plants, are found in fruits, vegetables, spices, tea, red wine, or beer [30,31]. They are suitable in chemoprevention because *in vitro* and *in vivo* research has demonstrated that they can sensitize TRAIL-resistant cancer cells and induce apoptosis [32,33,34,35]. Flavonoids are subdivided into flavones, isoflavones, flavanones, flavonols, chalcones, and anthocyanidins.

**Figure 1:**
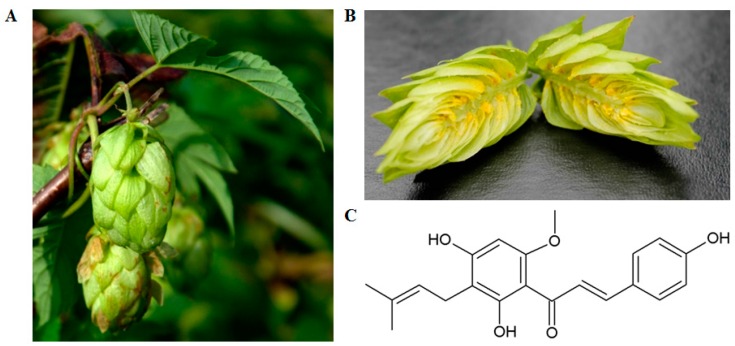
(**A**) Female strobiles [38]; (**B**) section through the mature strobili [39]; and (**C**) structure of xanthohumol.

Chalcones are the most structurally diverse groups of flavonoids [36]. One of them is xanthohumol, the principal prenylated chalcone (3’-[3,3-dimethyl allyl]-2’,4’-4-trihydroxy-6’-methoxychalcone) from the female inflorescences of the hop (*Humulus lupulus* L.). Xanthohumol is secreted as part of the yellowish substance named lupulin obtained from glands of the strobiles of the hop plant [37] (Figure 1B). Female strobiles are shown in Figure 1A and the structure of the studied compound is presented in Figure 1C.

Hop cones are an essential raw material used in beer brewing. It has been shown that xanthohumol has a broad spectrum of antibacterial, antioxidant, anticancer, chemopreventive, and anti-inflammatory properties [37,40,41,42,43,44,45]. We studied apoptotic and cytotoxic effects of TRAIL in combination with xanthohumol on androgen-sensitive human prostate adenocarcinoma cells (LNCaP). We have shown that xanthohumol can enhance apoptosis induced by TRAIL in cancer cells [42,43]. After treatment of prostate cancer cells with TRAIL and/or xanthohumol we analyzed the expression of proteins involved in apoptosis. We are the first to report the molecular mechanism by which xanthohumol in combination with TRAIL has sensitized TRAIL-resistant LNCaP cells leading to apoptosis.

## 2. Results

### 2.1. Cytotoxic and Apoptotic Activities of Tumor Necrosis Factor-Related Apoptosis-Inducing Ligand (TRAIL) in Androgen-Sensitive Human Prostate Adenocarcinoma Cells (LNCaP)

**Figure 2:**
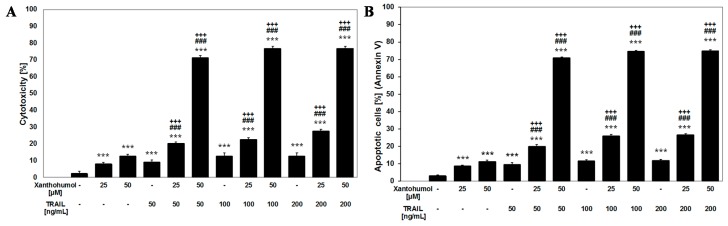

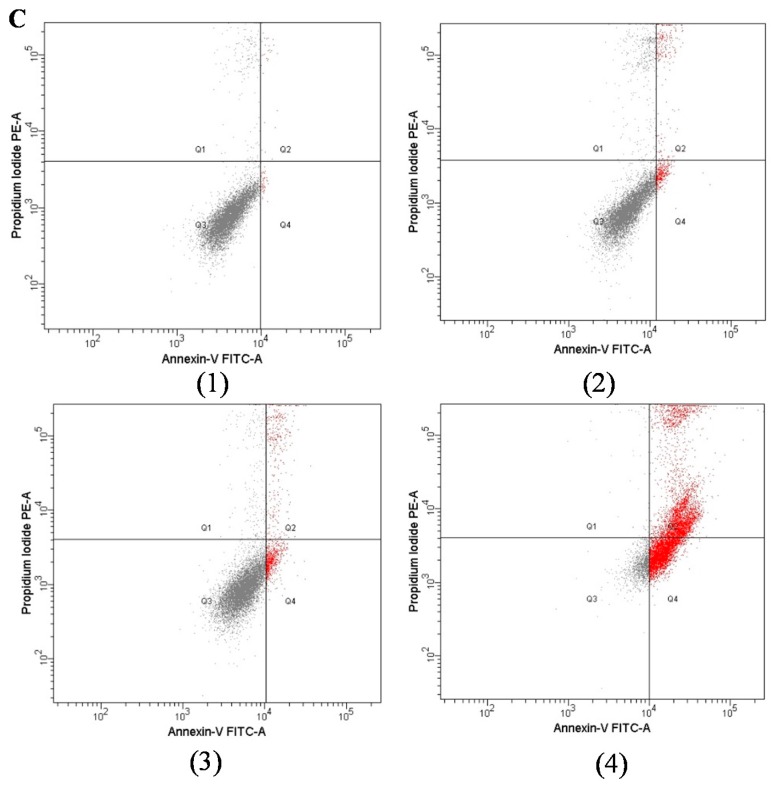
Cytotoxic and apoptotic effects of TRAIL in combination with xanthohumol in LNCaP cancer cells. The values represent mean ± SD of three independent experiments performed in quadruplicate (*n* = 12), *** *p* < 0.001 compared with control, ^###^ *p* < 0.001 compared with xanthohumol, ^+++^ *p* < 0.001 compared with TRAIL. (**A**) Cytotoxic effect of TRAIL in combination with xanthohumol in LNCaP cells; (**B**) apoptotic effect of TRAIL in combination with xanthohumol in LNCaP cells; (**C**) representative histograms: (**1**) control cells; (**2**) cells incubated with 100 ng/mL TRAIL; (**3**) cells incubated with 50 µM xanthohumol; (**4**) cells incubated with 100 ng/mL TRAIL + 50 µM xanthohumol. Red dots show apoptotic cells and gray dots live cells.

In our study recombinant human TRAIL in a soluble form was used. TRAIL induced cytotoxicity from 9.01% ± 1.35% to 12.57% ± 1.85% on LNCaP cells in a concentration of 50–100 ng/mL after a 24-h incubation. The cytotoxicity was determined by 3-(4,5-dimethylthiazol-2-yl)-2,5-diphenyltetrazolium bromide tetrazolium reduction assay (MTT). Apoptotic effect of 50–100 ng/mL TRAIL in LNCaP cells analyzed by flow cytometry ranged from 9.50% ± 0.97% to 11.46% ± 0.78%. Use of TRAIL in a concentration of 200 ng/mL did not significantly affect the cytotoxicity and apoptosis as compared to a concentration of 100 ng/mL (Figure 2A,B1). We obtained the results using the MTT assay and a flow cytometry which confirmed resistance of LNCaP cells to apoptosis mediated by TRAIL.

### 2.2. Cytotoxic and Apoptotic Activities of Xanthohumol in LNCaP Cancer Cells

The cytotoxic effect of xanthohumol against LNCaP cells depends on a concentration of the tested compound. Xanthohumol induced cytotoxicity equal 7.85% ± 1.00% in a concentration of 25 µM and 12.56% ± 1.21% in a concentration of 50 µM in LNCaP cells after a 24-h incubation. The results of cytotoxicity determined by MTT assay correlated with the percentage of apoptotic cells detected using flow cytometer (8.53% ± 0.60% to 11.10% ± 0.84% apoptosis in LNCaP cells after 24 h incubation with 25 and 50 µM of xanthohumol, respectively). Xanthohumol showed weak apoptotic and cytotoxic effects against LNCaP cells.

In our study we used 50 µM xanthohumol, which is more cytotoxic than 50 ng/mL TRAIL and comparably cytotoxic to 100 and 200 ng/mL TRAIL (because about 12.56% ± 1.21% cytotoxicity of 50 µM xanthohumol is compared to 12.51% ± 2.02% cytotoxicity of 100 ng/mL TRAIL and respectively 11.10% ± 0.84% apoptosis of 50 µM xanthohumol is compared to 12.57% ± 1.85% apoptosis of 100 ng/mL TRAIL). The results obtained from lactate dehydrogenase assay (LDH) and flow cytometer showed that 50 µM of xanthohumol caused apoptotic effect.

### 2.3. Apoptotic and Cytotoxic Activities of TRAIL in Combination with Xanthohumol on LNCaP

We examined the apoptotic and cytotoxic effects of TRAIL in combination with xanthohumol on LNCaP cancer cells. The cytotoxicity of 100 ng/mL TRAIL in combination with 25 and 50 µM xanthohumol in LNCaP cells was significantly increased to (22.48% ± 1.09%)–(76.58% ± 1.45%) of cell death (Figure 2A). Figure 2B1 demonstrates the percentage of apoptotic cells stained with Αnnexin V-FITC and analyzed by flow cytometry ((25.94% ± 0.95%)–(74.50% ± 0.57%) apoptosis in LNCaP cells after incubation with 100 ng/mL TRAIL in combination with 25–50 µM xanthohumol).

### 2.4. Necrotic Effect of TRAIL in Combination with Xanthohumol on LNCaP Cancer Cells

**Figure 3:**
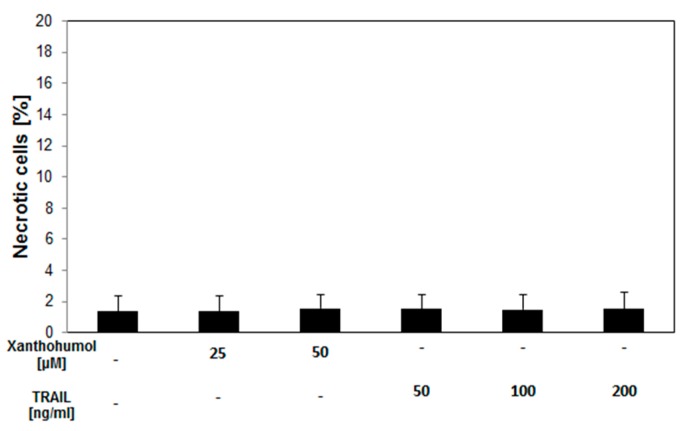
Necrotic effect of TRAIL and/or xanthohumol in LNCaP cancer cells. The percentage of necrotic cells was measured by lactate dehydrogenase assay (LDH) cytotoxicity assay. The values represent mean ± standard deviation (SD) of three independent experiments performed in quadruplicate (*n* = 12). No statistically significant differences were shown.

Co-incubation with 25 µM xanthohumol and 50–200 ng/mL TRAIL gives an additive response in cytotoxicity and apoptosis. An over-additive effect was detectable using 50 µM xanthohumol in combination with 50–200 ng/mL TRAIL. The necrotic cell death percentage of LNCaP cells treated with 25–50 µM xanthohumol and 50–200 ng/mL TRAIL examined by LDH assay was (1.43% ± 1.14%)–(1.60% ± 1.02%) and was not significant compared to the control (Figure 3).

It was shown that 50 µM of xanthohumol caused necrosis in 1.56% ± 0.90% measured by LDH assay but it was not significant compared to the control (Figure 3). In turn, TRAIL in tested concentrations did not cause necrosis and cytolysis in LNCaP cells marked by measuring lactate dehydrogenase in LDH assay ((1.54% ± 0.94%)–(1.53% ± 1.1%) necrosis was not significant compared to the control).

### 2.5. Effects of Xanthohumol on DR4/TRAIL-R1 and DR5/TRAIL-R2 Expression on Surface of LNCaP Cancer Cells

**Figure 4:**
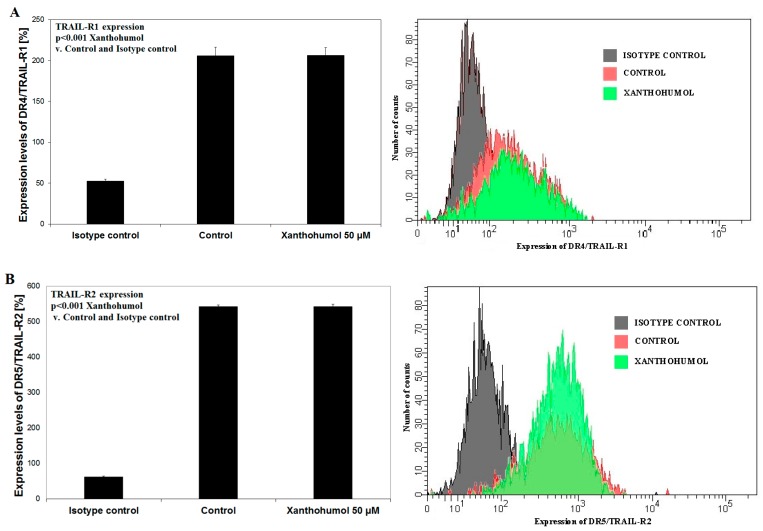
Effect of xanthohumol on death receptors expression on surface of LNCaP cancer cells. The expression of (**A**) DR4/TRAIL-R1 and (**B**) DR5/TRAIL-R2 on cancer cells together with representative histograms determined by flow cytometry. The values represent mean ± SD of three independent experiments performed in quadruplicate (*n* = 12). Statistical significance has not been shown for the results compared to the control.

The sensitivity of cancer cells to TRAIL is determined, *inter alia*, by expression of death receptors DR4/TRAIL-R1 and/or DR5/TRAIL-R2. Reduced expression of “death receptors” or gene mutations for these receptors leads to TRAIL-resistant cancer cells. In our study, LNCaP cells were incubated for 24 h with xanthohumol in a concentration of 50 µM. Prenylated chalcone did not affect the expression of death receptors TRAIL-R2 nor TRAIL-R1 on the surface of LNCaP cancer cells, which was evaluated using a flow cytometer (Figure 4).

### 2.6. Effects of TRAIL and/or Xanthohumol on the Mitochondrial Membrane Potential in LNCaP Cancer Cells

**Figure 5:**
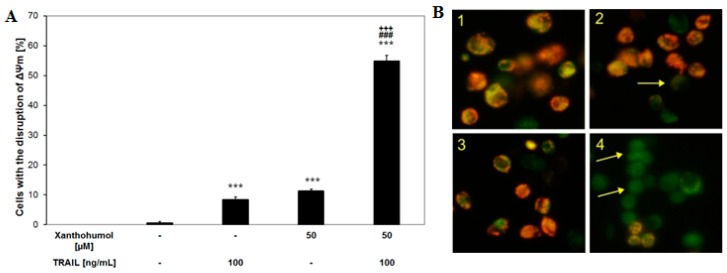
Mitochondrial membrane potential (ΔΨm) in LNCaP cancer cells. (**A**) TRAIL in combination with xanthohumol caused loss of ΔΨm in LNCaP cells. The values represent the mean ± SD of three independent experiments performed in duplicate *n* = 6 (*** *p* < 0.001 compared with control, ^###^ *p* < 0.001 compared with xanthohumol and ^+++^ *p* < 0.001 compared with TRAIL); (**B**) The loss of ΔΨm in cancer cells was evaluated by fluorescent microscopic analysis of DePsipher staining: (**1**) control cells; (**2**) cells incubated with 100 ng/mL TRAIL; (**3**) cells incubated with 50 µM xanthohumol; and (**4**) cells incubated with 100 ng/mL TRAIL + 50 µM xanthohumol. In cells with intact cell membrane aggregates of DePsipher emitting red fluorescence are formed in mitochondria. Green fluorescence is evidence of the monomeric form of the DePsipher molecule that is present in the cytosol after mitochondrial membrane depolarization (indicated by arrows); magnification 200×.

The loss of the mitochondrial membrane potential is one of the first intracellular changes leading to apoptosis. However, in some cell lines extrinsic signal induced by TRAIL binding to its “death receptor” is not sufficient for apoptosis. The amplification signal involvement by intrinsic (mitochondrial) pathway is necessary to complete the apoptotic process. The incubation of LNCaP cells with 100 ng/mL TRAIL or 50 µM xanthohumol alone caused little effect on ΔΨm (8.4 ± 1.0 and 11.2 ± 0.7, respectively). The combination of TRAIL with xanthohumol augmented the loss of ΔΨm in a large percentage of cells (54.9% ± 1.9%) and induced a significant disruption of ΔΨm (Figure 5A). The changes of ΔΨm in LNCaP cells after co-treatment with TRAIL and xanthohumol were evaluated using DePsipher staining by fluorescence microscopy (Figure 5B). These results demonstrated the engagement of intrinsic apoptotic pathway in LNCaP cells after the treatment with TRAIL and xanthohumol.

### 2.7. Effect of TRAIL in Combination with Xanthohumol on the Protein Expression in LNCaP Cells

In the process of apoptosis intracellular proteins are engaged. In this study we have shown for the first time that xanthohumol modulated TRAIL-resistance of LNCaP cancer cells to programmed cell death by activation and/or inhibition of the intracellular proteins. LNCaP cancer cells were treated with 100 ng/mL TRAIL and/or 25 µM xanthohumol for 2 and 8 h and the expression of proteins was determined by Western blotting.

**Figure 6:**
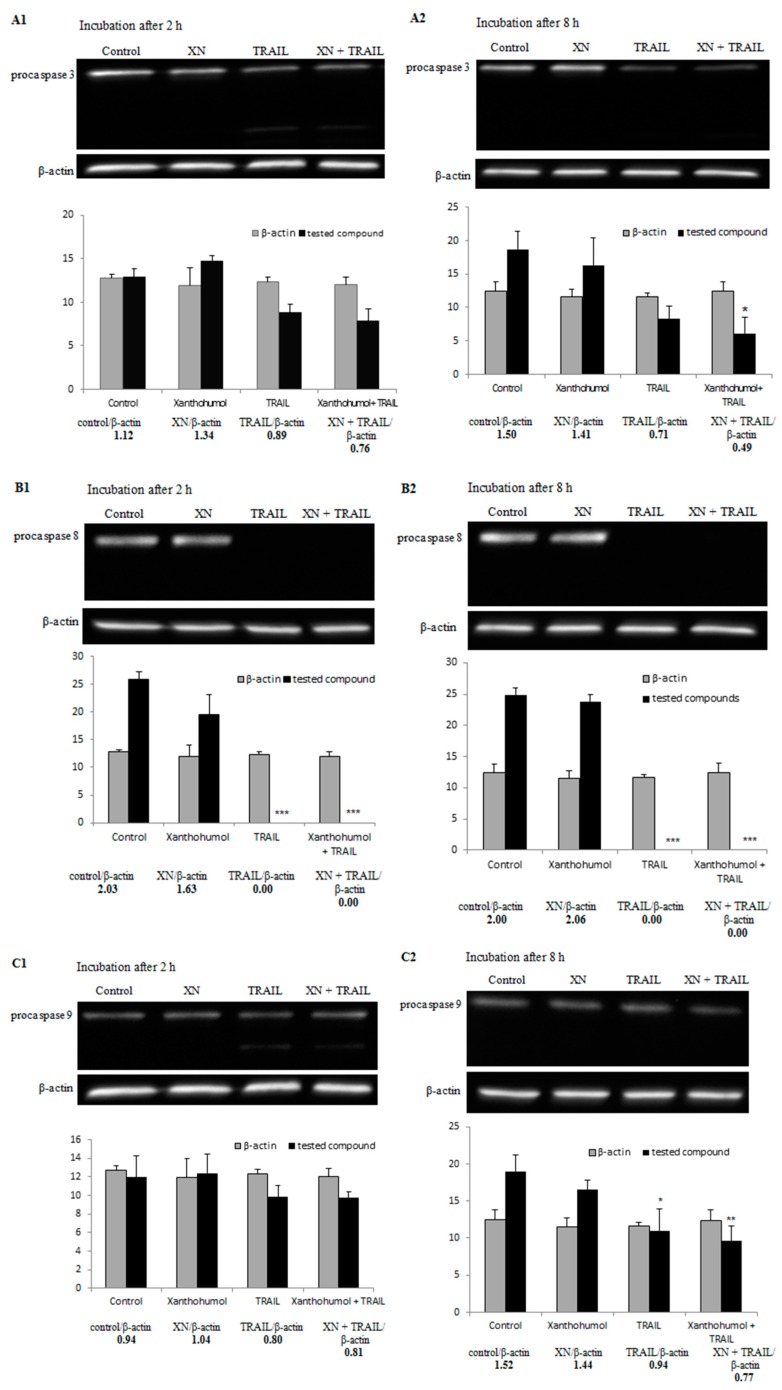

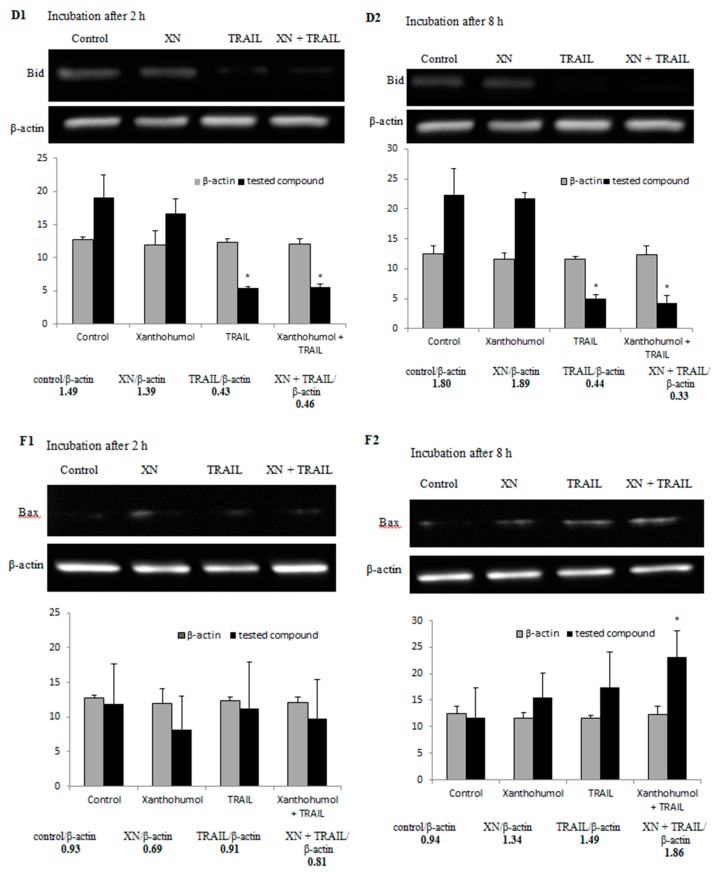
Effect of TRAIL and/or xanthohumol on the protein expression in LNCaP cells. Cancer cells were treated with 50 µM xanthohumol and/or 100 ng/mL TRAIL for 2 or 8 h. The expressions of (**A**–**C**) caspases (-3, -8, -9) and (**D**–**F**) Bcl-2 family members (Bid, Bax, Bcl-xL) were measured by the Western blot technique. The results are expressed as means ± SD obtained from three independent experiments. The β-actin was used as a control to show equal loading of proteins. The data were normalized to the control and β-actin level. A statistical significance of the differences between the treatment and control results is marked with * *p* < 0.05, ** *p* < 0.01, *** *p* < 0.001.

**Figure 7:**
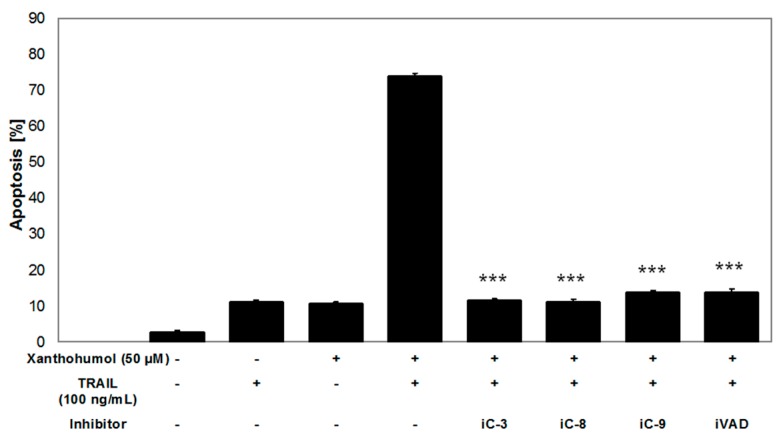
Inhibition of the apoptotic effect of TRAIL in combination with xanthohumol on LNCaP cells after application of caspase inhibitors: -3 (iC-3), -8 (iC-8), -9 (iC-9), and inhibitor of all caspases (iVAD). LNCaP cells were incubated with 100 ng/mL TRAIL and/or 50 µM xantohumol and/or 20 µM caspase inhibitors for 24 h. Apoptotic cells were detected using flow cytometer with Apoptotest-FITC (*** *p* < 0.001 compared with TRAIL in combination with xanthohumol).

Caspases, a family of cysteine proteases, play an important role in programmed cell death. In our study, xanthohumol alone did not cleave the examined caspases (-3, -8, -9). Here we have shown that TRAIL in combination with xanthohumol cleaved caspases-8, -9, and -3 (Figure 6A–C) after 2 and/or 8 h. In order to confirm that apoptosis induced by TRAIL in combination with xanthohumol is dependent on caspases, we used caspase inhibitors: caspase-3 inhibitor (iC-3, z-DEVD-fmk), caspase-8 inhibitor (iC-8, z-IETD-fmk), caspase-9 inhibitor (iC-9, z-LEHD-fmk), and pancaspase inhibitor (iVAD, z-VAD-fmk). The use of caspase inhibitors in combination with TRAIL and xanthohumol caused a significant inhibition of apoptosis in LNCaP cells from 73.9 ± 0.7 to 11.5 ± 0.6 with iC-3, 11.2 ± 0.6 with iC-8, 13.7 ± 0.6 with iC-9, and 13.9 ± 0.9 with iVAD (Figure 7). These results suggest that TRAIL-induced apoptosis in combination with xanthohumol extends extrinsic pathway involving caspases.

In our study, xanthohumol in a concentration of 25 and after 2 and 8 h incubation of LNCaP cancer cells alone did not affect the expression of proapoptotic proteins Bid and Bax. TRAIL alone and TRAIL used with xanthohumol induced activation of Bid and led to a significant increase of the expression of Bax after 2 and 8 h incubation (the results are shown in Figure 6D,E), respectively. In this paper we have also demonstrated that TRAIL in combination with xanthohumol decreased the expression of anti-apoptotic protein Bcl-xL after 8 h incubation (Figure 6F).

## 3. Discussion

Flavonoids which are isolated from vegetables, fruits, spices, green tea, red wine, and beer are extensively researched for their cancer chemopreventive potential [46]. Xanthohumol, a natural flavonoid derivative from hops, exhibits a broad spectrum of biological activities [47,48]. It causes cell cycle arrest or apoptotic effect in many cancer cell types [40,49,50,51]. In our study xanthohumol showed weak cytotoxic and apoptotic effects against LNCaP cells.

TRAIL (Apo2L/TRAIL), an important component of the immune defense, induces apoptosis in a variety of cancer cells without toxicity to normal cells [10,16]. In their previous study Szliszka *et al.* [43,52] demonstrated that LNCaP prostate cancer cells are resistant to TRAIL-induced apoptosis. Natural and synthetic flavonoids can sensitize cancer cells by increasing their susceptibility to TRAIL-induced apoptosis. Flavonoids (flavonols, isoflavones, chalcones) play an important role in prostate cancer chemoprevention for they enhance TRAIL-mediated apoptosis [52,53,54,55,56,57]. Recent studies have revealed that chalcones, such as butein, chalcone, isobavachalcone, licochalcone-A, and xanthohumol, in combination with TRAIL cause an increase in the percentage of cell death in prostate cancer cells [43]. In this study, we have confirmed that xanthohumol sensitized TRAIL-resistant human LNCaP cells.

Szliszka *et al.* [42] reported that xanthohumol (25 µM) significantly increased TRAIL-R2 protein levels on the surface of HeLa cervical cancer cells and caused a small effect on the TRAIL-R1 receptor expression after 24-h incubation. Szliszka *et al.* [52] also proved that LNCaP prostate cancer cells incubated with fisetin (a flavonol present in apples, grapes and strawberries) caused a significant increase of the expression of TRAIL-R1 in LNCaP cells. The TRAIL-R2 expression in prostate cancer cells was unaltered. Incubation of TRAIL-resistant LNCaP cells with 20 or 40 µM epigallocatechin-3-gallate (EGCG), a polyphenol found predominately in green tea, caused an increase of TRAIL-R1 expression in LNCaP cancer cells [58]. Another natural compound—resveratrol (polyphenol belonging to stilbens and isolated from *Veratrum grandiflorum*, *Polygonum cuspidatum*, and red grapes) induced expression of receptors TRAIL-R1 and TRAILR2 in LNCaP cells [59]. We analyzed the expression of death receptors in LNCaP cells after 24-h treatment with 50 µM of xanthohumol. Our test demonstrated that xanthohumol did not influence the expression of both death receptors.

One of the first intracellular changes in apoptosis is a disruption of the mitochondrial membrane potential. Our previous study on LNCaP prostate cancer cells confirmed the role of mitochondrial disruption due to chalcones in TRAIL-mediated apoptotic pathway [57]. Therefore, we analyzed the mitochondrial membrane potential after incubation with TRAIL and/or xanthohumol. TRAIL and xanthohumol co-treatment induced a significant reduction of mitochondrial membrane potential in a large percentage of LNCaP cancer cells compared to each of these agents alone. In comparison, Ca Ski cervical cancer cells treated with xanthohumol at the concentrations of 20, 30, and 40 µM caused decreases in the percentage of viable cells with high ΔΨm from 99.0% to 55.4% [60].

Caspases play an essential role in apoptosis. They are produced as inactive procaspases and cleaved into active forms. Caspases are classified by their mechanism in apoptosis as initiator caspases (-8, -9, -10) and executioner caspases (-3, -6, -7) [10,16,52]. In our study xanthohumol alone did not cleave the caspases-3, -8, and -9. Similar results were obtained by Delmulle *et al.* [61], who applied 200 µM xanthohumol or other prenylflavonoids (isoxanthohumol, 8-prenylnaringenin, and 6-prenylnaringenin) and incubated PC-3 prostate cancer cells with prenylflavonoids for 15, 30, 60, 90, and 120 min. They demonstrated that the investigated compounds did not activate caspase-3. In turn, Deeb *et al.* [62] showed that incubation of C4-2 and PC-3 prostate cancer cells for 72 h with xanthohumol in concentrations of 20 and 40 µM completely activated procaspase-8, -9, and -3. In our study, combination of TRAIL with xanthohumol activates caspases-3, -8, and -9 in LNCaP cells. Similar observations from *in vitro* studies on prostate cancer cells have been described by Siddiqui *et al.* [58]. The researchers incubated TRAIL-resistant LNCaP cells with 20–40 µM EGCG and/or 100 ng/mL TRAIL and demonstrated that the combination of TRAIL with EGCG activates initiator caspases (-8, -9) and effector caspases (-3, -6) more than each agent alone.

Intrinsic and extrinsic pathways in apoptosis are joined by a proapoptotic protein Bid. Caspase-8 cleaves Bid into tBid which is translocated to mitochondria where it activates other proapoptotic proteins, such as Bax and Bak to their oligomer forms. This causes a release of cytochrome c from mitochondria. Bax and Bak proteins are important in TRAIL-induced apoptosis. Cells lacking Bax and/or Bak are resistant to apoptosis [63,64]. In our study, xanthohumol used alone did not affect the expression of proapoptotic proteins Bid and Bax of LNCaP cancer cells. In turn, TRAIL alone and TRAIL used with xanthohumol induced activation of Bid and led to a significant increase of the expression of Bax after 2 and 8 h incubation, respectively. Similar results were obtained by Siddiqui *et al.*, who incubated TRAIL-resistant LNCaP cells with TRAIL and/or EGCG. They also demonstrated that EGCG and TRAIL combined together increased the levels of proapoptotic Bak and Bax [58]. In turn, resveratrol used alone resulted in translocation of Bax to mitochondria at 2 and 4 h in LNCaP cells [59].

One of the reasons for cancer cell resistance to apoptosis mediated by TRAIL is the overexpression of antiapoptotic proteins BcL-2 and/or Bcl-xL [65,66]. Jiang *et al.* [67] showed that xanthohumol in the concentration of 10 µM inhibited the expression of Bcl-xL at the messenger RNA level in PANC-1 pancreatic cancer cells after 24 h incubation. We have also demonstrated that TRAIL in combination with xanthohumol decreased the expression of anti-apoptotic protein Bcl-xL after 8 h incubation. A similar result was obtained by Siddiqui *et al.* [58] after the incubation of LNCaP prostate cancer cells with TRAIL and EGCG, and also by Kim *et al.* [68] after treatment of DU-145 prostate cancer cells with TRAIL and quercetin.

## 4. Materials and Methods

### 4.1. Cell Culture

Human prostate cancer LNCaP cell line was purchased from DSMZ (Deutsche Sammlung von Mikroorganismen und Zellkulturen) GmbH-German Collection of Microorganism and Cell Cultures (Braunschweig, Germany). The LNCaP cells were maintained in monolayer cultures in RPMI 1640 containing 10% fetal bovine serum (FBS), 100 IU/mL penicillin, and 100 µg/mL streptomycin. The cancer cells were grown at 37 °C in an atmosphere with 5% CO_2_ and 100% humidity. All reagents for cell culture were obtained from ATCC (American Type Culture Collection, Manassas, VA, USA).

### 4.2. Reagents

Soluble recombinant human TRAIL (rhsTRAIL) was obtained from PeproTech Inc. (Rocky Hill, NJ, USA). Xanthohumol was purchased from Alexis Biochemicals (San Diego, CA, USA) and dissolved in DMSO (50 mM) to a final concentration of 0.1% (*v*/*v*) in the culture media. The purity of tested xanthohumol was ≥98% (HPLC).

### 4.3. Cytotoxicity Assay

Cytotoxicity was measured by the 3-(4,5-dimethylthiazol-2-yl)-2,5-diphenyltetrazolium bromide (MTT) assay [42]. Cells (1 × 10^5^/mL) were seeded into each well of a 96-well plate for 48 h. Then the cells were treated with xanthohumol at concentrations of 25–50 µM and/or TRAIL at final concentrations of 50–200 ng/mL for 24 h. After this time supernatants were removed and 180 µL culture medium and 20 µL MTT solution added to reach a final concentration of 1.1 mM. The cells were incubated for 4 h at 37 °C in an atmosphere containing 5% CO_2_ at 100% relative humidity. The resulting blue formazan crystals were dissolved in DMSO. The controls were native cells and medium alone. Reagents were obtained from Sigma Chemical Company (St. Louis, MO, USA). Spectrophotometric absorbance was measured at 550 nm wavelength with use of an Eon microplate reader (Bio-Tek Instruments Inc., Winooski, VT, USA). The cytotoxicity was calculated according to the formula: percent cytotoxicity (cell death) = [1 − (absorbance of experimental wells/absorbance of control wells)] × 100% [42,43,69].

### 4.4. Lactate Dehydrogenase Release Assay

Lactate dehydrogenase (LDH) is a stable cytosolic enzyme released into the supernatant as a result of cell membrane damage and cell lysis. The increase of LDH activity in the supernatant was correlated with the percentage of necrotic cells. LDH activity measurement was performed using the cytotoxicity assay kit from Roche Diagnostics GmbH (Mannheim, Germany). The LNCaP cells (1 × 10^6^ cells/ mL) were treated with TRAIL (50–200 ng/mL) and/or xanthohumol (25 and 50 µM). Maximal LDH release was obtained after the treatment of control cells with 1% solution of Triton X-100 (Sigma Chemical Company) for 10 min at room temperature. The spectrophotometric absorbance was measured at 490 nm using the Eon microplate reader. The necrotic cell percentage was calculated using the formula: (sample value/maximal release) × 100% [53,70].

### 4.5. Detection of Apoptotic Cell Death by Flow Cytometry

Apoptosis was measured by flow cytometry using the Apoptotest-FITC Kit with Annexin V (Dako, Glostrup, Denmark). Prostate cancer LNCaP cells (1 × 10^5^/mL) were seeded in 24-well plates for 48 h and then exposed to xanthohumol (25 and 50 µM) and/or TRAIL (50–200 ng/mL) for 24 h. After this time the cancer cells were washed twice with phosphate-buffered saline (PBS) solution and resuspended in binding buffer (500 µL). The cell suspension was incubated with Annexin V-FITC (5 µL) and propidium iodide (5 µL) for 10 min at room temperature in the dark. The population of Annexin V-positive cells was evaluated by flow cytometry (LSR II, Becton Dickinson Immunocytometry Systems, San Jose, CA, USA) [56,71].

### 4.6. Flow Cytometric Analysis of Death Receptor Expression on the Cancer Cell Surface

The expression of death receptors (DR4/TRAIL-R1 and DR5/TRAIL-R2) was determined using a LSR II flow cytometer (San Jose, CA, USA). Cells were adherent in a 24-well plate for 24 h and incubated with xanthohumol (50 µM). After this time the cells were harvested using trypsin solution and ethylenediaminetetraacetic acid (EDTA). The cells were washed twice with PBS solution and resuspended with PBS containing 0.5% bovine serum albumin (BSA). Cells were Fc-blocked for 15 min at room temperature. Then the cells were incubated with 10 µL monoclonal antibody anti-DR4/TRAIL-R1 or anti-DR5/TRAIL-R2 conjugated with phycoerythrin (PE) (R and D Systems, Minneapolis, MN, USA) at 4 °C for 45 min. Finally, the cells were washed with PBS and analyzed by flow cytometry. Cells in a separate tube treated with phycoerythrin-labelled mouse IgG1 or mouse IgG2B (R and D Systems) constituted the control sample (isotype control) [72,73].

### 4.7. Mitochondrial Depolarization Assay

The decreased mitochondrial membrane potential (ΔΨm) was evaluated with fluorescence microscopy using a DePsipher kit (R and D Systems). LNCaP cells (1 × 10^5^/mL) were seeded in a 24-well plate 24 h prior to the experiments. The cells were stimulated with TRAIL (100 ng/mL) and/or xanthohumol (50 µM) for 24 h. Then the LNCaP cells were washed with PBS and harvested by 0.25% trypsin solution. The cells were incubated in the dark with DePsipher (5,5′,6,6′-tetrachloro-1,1′,3,3′-tetraethyl-benzimidazolyl carbocyanin iodide) solution at a concentration of 5 µg/mL at 37 °C for 30 min. Then they were washed with reaction buffer with stabilizer, placed on a glass slide and covered with a glass coverslip. The total amount of 200 cells per sample was taken for analysis. The stained cells were observed under fluorescence inverted microscope IX-51 (Olympus, Tokyo, Japan), using filter sets for FITC and TRITC. DePsipher accumulated in mitochondria depending on the potential. In cells unchanged from the mitochondrial membrane potential dye aggregates exhibit red fluorescence (590 nm). At low mitochondrial potential (the mitochondrial membrane depolarization) DePsipher monomeric forms appear in the cytosol and emit green fluorescence (530 nm). The cells were counted each time from the representative area containing 100 cells and cells with mitochondrial membrane depolarization were expressed as percentage of total cells [52,54,56,69].

### 4.8. Western Blotting

LNCaP cells were adhered in Petri dishes (3.5 cm diameter) for 48 h and then treated with 100 ng/mL TRAIL and/or 25 µM xanthohumol. The cells were washed twice with cold PBS and lysed in a Ripa Buffer with Protease Inhibitor Cocktail (Sigma-Aldrich, St. Louis, MO, USA). Protein samples (20 µg) were separated on 12% sodium dodecyl sulfate (SDS)-polyacrylamide gels using a Mini Trans-Blot (BioRad, Hercules, CA, USA). Proteins were transferred onto a Whatman nitrocellulose membrane (Labo Plus, Warsaw, Poland). The membrane was blocked in 5% non-fat dry milk for 1 h in room temperature and incubated at 4 °C overnight with the following primary antibodies (1:1000 dilutions): anti-procaspase 3, anti-procaspase 8, anti-procaspase 9, anti-Bid, anti-cytochrome *c*, anti-Bax, anti-Bad, anti-Bcl-2, anti-Bcl-xL, and anti-β-actin (Cell Signaling Technology, Danvers, MA, USA). After thorough washing, the membranes were incubated with secondary antibodies conjugated with horseradish peroxidase (HRP) for 1 h at room temperature. Then the membranes were incubated for 1 min with a chemiluminescence detection chromogen Phototope^®^-HRP Western Blot Detection System (Cell Signaling Technology). The detection of chemiluminescence and photographic documentation was performed using ChemiDoc™ XRS+ Systems with Image Lab™ software (BioRad).

### 4.9. Apoptosis Inhibition Using Caspase Inhibitors

In order to determine whether TRAIL and/or xanthohumol—induced apoptosis is dependent on caspases, the following caspase inhibitors were used: caspase-3 inhibitor (z-DEVD-fmk), caspase-8 inhibitor (z-IETD-fmk), caspase-9 inhibitor (z-LEHD-fmk) and pancaspase inhibitor (z-VAD-fmk). The caspase inhibitors were obtained from R and D Systems [74]. The results of our earlier experiments (data not shown) confirmed that caspase inhibitors used alone did not reduce the viability of LNCaP cells (MTT assay) and did not induce apoptosis or necrosis of LNCaP cells (Apoptotest-FITC) [73]. LNCaP cells were incubated with 100 ng/mL TRAIL and/or 50 µM xantohumol and/or 20 µM caspase inhibitors for 24 h. Apoptotic cells were detected using flow cytometer LSR II with Apoptotest-FITC.

### 4.10. Statistical Analysis

The results are presented as means ± SD obtained from three separate experiments done in duplicate or quadruplicate (*n* = 6 or 12). Statistical significance was evaluated using ANOVA or Student’s *t*-test. The *p*-values < 0.05 were considered significant. For statistical calculation we used Microsoft Excel 2007 and StatSoft Statistica version 10.0 (Cracow, Poland). In order to perform the analysis of western blot technique results Image J software (Bethesda, MD, USA) was used. This program calculates what percentage of the total density each band has. The results are expressed as means ± SD obtained from three independent experiments. We normalized the data to the control and the β-actin level.

## 5. Conclusions

Xanthohumol supports apoptotic and cytotoxic activities of TRAIL on prostate LNCaP cancer cells. This prenylated chalcone sensitizes cancer cells to TRAIL-mediated apoptosis through modulation of extrinsic and intrinsic apoptotic pathways. The potential apoptotic mechanism, after application of TRAIL in combination with xanthohumol, is most likely associated with the activation of caspases-3, -8, -9, with the activation of Bid, with the increase of the expression of Bax, the decrease of the expression of Bcl-xL, and also with the decrease of mitochondrial potential in LNCaP cells. Xanthohumol sensitizes LNCaP cancer cells to apoptosis induced by TRAIL and, therefore, it can enhance the natural mechanisms of anti-tumor immunity.

## References

[1] Wiley S.R., Schooley K., Smolak P.J., Din W.S., Huang C.P., Nicholl J.K., Sutherland G.R., Smith T.D., Rauch C., Smith C.A. (1995). Identification and characterization of a new member of the TNF family that induces apoptosis. Immunity.

[2] Pitti R.M., Marsters S.A., Ruppert S., Donahue C.J., Moore A., Ashkenazi A. (1996). Induction of apoptosis by Apo-2 ligand, a new member of the tumor necrosis factor cytokine family. J. Biol. Chem..

[3] Secchiero P., Gonelli A., Corallini F., Ceconi C., Ferrari R., Zauli G. (2010). Metalloproteinase 2 cleaves *in vitro* recombinant TRAIL: Potential implications for the decreased serum levels of TRAIL after acute myocardial infarction. Atherosclerosis.

[4] Halaas O., Vik R., Ashkenazi A., Espevik T. (2000). Lipopolysaccharide induces expression of Apo-2 ligand/TRAIL in human monocytes and macrophages. Scand. J. Immunol..

[5] Liu S., Yu Y., Zhang M., Wang W., Cao X. (2001). The involvement of TNF-α-related apoptosis-inducing ligand in the enhanced cytotoxicity of IFN-β-stimulated human dendritic cells to tumor cells. J. Immunol..

[6] Johnsen A.C., Haux J., Steinkjer B., Nonstad U., Egeberg K., Sundan A., Ashkenazi A., Espevik T. (1999). Regulation of Apo-2 ligand/TRAIL expression in NK cells-involvement in NK cell-mediated cytotoxicity. Cytokine.

[7] Thomas W.D., Hersey P. (1998). TNF-related apoptosis-inducing ligand (TRAIL) induces apoptosis in Fas ligand-resistant melanoma cells and mediates CD4 T cell killing of target cells. J. Immunol..

[8] Ashkenazi A., Dixit V.M. (1998). Death receptors: Signaling and modulation. Science.

[9] Zhang X.D., Nguyen T., Thomas W.D., Sanders J.E., Hersey P. (2000). Mechanisms of resistance of normal cells to TRAIL induced apoptosis vary between different cell types. FEBS Lett..

[10] Almasan A., Ashkenazi A. (2003). Apo2L/TRAIL: Apoptosis signaling, biology, and potential for cancer therapy. Cytokine Growth Factor Rev..

[11] Pan G., O′Rourke K., Chinnaiyan A.M., Gentz R., Ebner R., Ni J., Dixit V.M. (1997). The receptor for the cytotoxic ligand TRAIL. Science.

[12] Pan G., Ni J., Wei Y.F., Yu G., Gentz R., Dixit V.M. (1997). An antagonist decoy receptor and a death domain-containing receptor for TRAIL. Science.

[13] Degli-Esposti M.A., Smolak P.J., Walczak H., Waugh J., Huang C.P., DuBose R.F., Goodwin R.G., Smith C.A. (1997). Cloning and characterization of TRAIL-R3, a novel member of the emerging TRAIL receptor family. J. Exp. Med..

[14] MacFarlane M., Ahmad M., Srinivasula S.M., Fernandes-Alnemri T., Cohen G.M., Alnemri E.S. (1997). Identification and molecular cloning of two novel receptors for the cytotoxic ligand TRAIL. J. Biol. Chem..

[15] Pennarun B., Meijer A., de Vries E.G., Kleibeuker J.H., Kruyt F., de Jong S. (2010). Playing the DISC: Turning on TRAIL death receptor-mediated apoptosis in cancer. Biochim. Biophys. Acta.

[16] Holoch P.A., Griffith T.S. (2009). TNF-related apoptosis-inducing ligand (TRAIL): A new path to anti-cancer therapies. Eur. J. Pharmacol..

[17] Horak P., Pils D., Haller G., Pribill I., Roessler M., Tomek S., Horvat R., Zeillinger R., Zielinski C., Krainer M. (2005). Contribution of epigenetic silencing of tumor necrosis factor-related apoptosis inducing ligand receptor 1 (DR4) to TRAIL resistance and ovarian cancer. Mol. Cancer Res..

[18] Sanlioglu A.D., Dirice E., Aydin C., Erin N., Koksoy S., Sanlioglu S. (2005). Surface TRAIL decoy receptor-4 expression is correlated with TRAIL resistance in MCF7 breast cancer cells. BMC Cancer.

[19] Griffith T.S., Lynch D.H. (1998). TRAIL: A molecule with multiple receptors and control mechanisms. Curr. Opin. Immunol..

[20] Krajewska M., Krajewski S., Banares S., Huang X., Turner B., Bubendorf L., Kallioniemi O.P., Shabaik A., Vitiello A., Peehl D. (2003). Elevated expression of inhibitor of apoptosis proteins in prostate cancer. Clin. Cancer Res..

[21] Sinicrope F.A., Penington R.C., Tang X.M. (2004). Tumor necrosis factor-related apoptosis -inducing ligand-induced apoptosis is inhibited by Bcl-2 but restored by the small molecule Bcl-2 inhibitor, HA 14–1, in human colon cancer cells. Clin. Cancer Res..

[22] Hinz S., Trauzold A., Boenicke L., Sandberg C., Beckmann S., Bayer E., Walczak H., Kalthoff H., Ungefroren H. (2000). Bcl-xl protects pancreatic adenocarcinoma cells against CD95- and TRAIL-receptor-mediated apoptosis. Oncogene.

[23] Du X., Xiang L., Mackall C., Pastan I. (2011). Killing of resistant cancer cells with low Bak by a combination of an antimesothelin immunotoxin and a TRAIL receptor 2 agonist antibody. Clin. Cancer Res..

[24] Gillissen B., Wendt J., Richter A., Müer A., Overkamp T., Gebhardt N., Preissner R., Belka C., Dörken B., Daniel P.T. (2010). Endogenous Bak inhibitors MCL-1 and Bcl-xL: Differential impact on TRAIL resistance in Bax-deficient carcinoma. J. Cell. Biol..

[25] Szliszka E., Krol W. (2011). The role of dietary polyphenols in tumor necrosis factor-related apoptosis inducing ligand (TRAIL)-induced apoptosis for cancer chemoprevention. Eur. J. Cancer Prev..

[26] Jemal A., Siegel R., Xu J., Ward E. (2010). Cancer statistics, 2010. CA Cancer J. Clin..

[27] Zhang S., Wang Y., Chen Z., Kim S., Iqbal S., Chi A., Ritenour C., Wang Y.A., Kucuk O., Wu D. (2013). Genistein enhances the efficacy of cabazitaxel chemotherapy in metastatic castration-resistant prostate cancer cells. Prostate.

[28] Kowalska M., Szliszka E., Król W. (2013). The pathway of tumor necrosis factor-related apoptosis inducing ligand (TRAIL) as a potential target in therapy of prostate cancer. Ann. Acad. Med. Siles.

[29] Sporn M.B., Dunlop N.M., Newton D.L., Smith J.M. (1976). Prevention of chemical carcinogenesis by vitamin A and its synthetic analogs (retinoids). Fed. Proc..

[30] Kozłowska A., Szostak-Wegierek D. (2014). Flavonoids-food sources and health benefits. Rocz. Panstw. Zakl. Hig..

[31] Toh J.Y., Tan V.M., Lim P.C., Lim S.T., Chong M.F. (2013). Flavonoids from fruit and vegetables: A focus on cardiovascular risk factors. Curr. Atheroscler. Rep..

[32] Abdelhamed S., Yokoyama S., Hafiyani L., Kalauni S.K., Hayakawa Y., Awale S., Saiki I. (2013). Identification of plant extracts sensitizing breast cancer cells to TRAIL. Oncol. Rep..

[33] Bronikowska J., Szliszka E., Jaworska D., Czuba Z.P., Krol W. (2012). The coumarin psoralidin enhances anticancer effect of tumor necrosis factor-related apoptosis-inducing ligand (TRAIL). Molecules.

[34] Yoshida T., Konishi M., Horinaka M., Yasuda T., Goda A.E., Taniguchi H., Yano K., Wakada M., Sakai T. (2008). Kaempferol sensitizes colon cancer cells to TRAIL-induced apoptosis. Biochem. Biophys. Res. Commun..

[35] Jin C.Y., Park C., Moon S.K., Kim G.Y., Kwon T.K., Lee S.J., Kim W.J., Choi Y.H. (2009). Genistein sensitizes human hepatocellular carcinoma cells to TRAIL-mediated apoptosis by enhancing Bid cleavage. Anticancer Drugs.

[36] Zhang E.H., Wang R.F., Guo S.Z., Liu B. (2013). An update on antitumor activity of naturally occurring chalcones. Evid. Based Complement. Altern. Med..

[37] Stevens J.F., Page J.E. (2004). Xanthohumol and related prenylflavonoids from hops and beer: To your good health!. Phytochemistry.

[38] Chmiel [hops]. http://photo.podsiadly.info/2009/08/31/chmiel/.

[39] Hops Blog. http://www.novascotiahopsblog.com/2013_08_01_archive.html.

[40] Dorn C., Weiss T.S., Heilmann J., Hellerbrand C. (2010). Xanthohumol, a prenylated chalcone derived from hops, inhibits proliferation, migration and interleukin-8 expression of hepatocellular carcinoma cells. Int. J. Oncol..

[41] Henderson M.C., Miranda C.L., Stevens J.F., Deinzer M.L., Buhler D.R. (2000). *In vitro* inhibition of human P450 enzymes by prenylated flavonoids from hops, *Humulus lupulus*. Xenobiotica.

[42] Szliszka E., Jaworska D., Kłósek M., Czuba Z.P., Król W. (2012). Targeting death receptor TRAIL-R2 by chalcones for TRAIL-induced apoptosis in cancer cells. Int. J. Mol. Sci..

[43] Szliszka E., Czuba Z.P., Mazur B., Sedek L., Paradysz A., Krol W. (2009). Chalcones enhance TRAIL-induced apoptosis in prostate cancer cells. Int. J. Mol. Sci..

[44] Kang Y., Park M.A., Heo S.W., Park S.Y., Kang K.W., Park P.H., Kim J.A. (2013). The radio-sensitizing effect of xanthohumol is mediated by STAT3 and EGFR suppression in doxorubicin-resistant MCF-7 human breast cancer cells. Biochim. Biophys. Acta.

[45] Liu M., Hansen P.E., Wang G., Qiu L., Dong J., Yin H., Qian Z., Yang M., Miao J. (2015). Pharmacological profile of xanthohumol, a prenylated flavonoid from hops (*Humulus lupulus*). Molecules.

[46] Park E.J., Pezzuto J.M. (2012). Flavonoids in cancer prevention. Anticancer Agents Med. Chem..

[47] Gerhauser C., Alt A., Heiss E., Gamal-Eldeen A., Klimo K., Knauft J., Neumann I., Scherf H.R., Frank N., Bartsch H. (2002). Cancer chemopreventive activity of xanthohumol, a natural product derived from hop. Mol. Cancer Ther.

[48] Gerhäuser C. (2005). Beer constituents as potential cancer chemopreventive agents. Eur. J. Cancer.

[49] Blanquer-Rosselló M.M., Oliver J., Valle A., Roca P. (2013). Effect of xanthohumol and 8-prenylnaringenin on MCF-7 breast cancer cells oxidative stress and mitochondrial complexes expression. J. Cell. Biochem..

[50] Drenzek J.G., Seiler N.L., Jaskula-Sztul R., Rausch M.M., Rose S.L. (2011). Xanthohumol decreases Notch1 expression and cell growth by cell cycle arrest and induction of apoptosis in epithelial ovarian cancer cell lines. Gynecol. Oncol..

[51] Delmulle L., Bellahcène A., Dhooge W., Comhaire F., Roelens F., Huvaere K., Heyerick A., Castronovo V., de Keukeleire D. (2006). Anti-proliferative properties of prenylated flavonoids from hops (*Humulus lupulus* L.) in human prostate cancer cell lines. Phytomedicine.

[52] Szliszka E., Helewski K.J., Mizgala E., Krol W. (2011). The dietary flavonol fisetin enhances the apoptosis-inducing potential of TRAIL in prostate cancer cells. Int. J. Oncol..

[53] Szliszka E., Czuba Z.P., Mertas A., Paradysz A., Krol W. (2013). The dietary isoflavone biochanin-a sensitizes prostate cancer cells to TRAIL-induced apoptosis. Urol. Oncol..

[54] Szliszka E., Zydowicz G., Janoszka B., Dobosz C., Kowalczyk-Ziomek G., Krol W. (2011). Ethanolic extract of brazilian green propolis sensitizes prostate cancer cells to TRAIL-induced apoptosis. Int. J. Oncol..

[55] Ismail B., Fagnere C., Limami Y., Ghezali L., Pouget C., Fidanzi C., Ouk C., Gueye R., Beneytout J.L., Duroux J.L. (2015). 2′-Hydroxy-4-methylsulfonylchalcone enhances TRAIL-induced apoptosis in prostate cancer cells. Anticancer Drugs.

[56] Szliszka E., Krol W. (2011). Soy isoflavones augment the effect of TRAIL-mediated apoptotic death in prostate cancer cells. Oncol. Rep..

[57] Szliszka E., Czuba Z.P., Mazur B., Paradysz A., Krol W. (2010). Chalcones and dihydrochalcones augment TRAIL-mediated apoptosis in prostate cancer cells. Molecules.

[58] Siddiqui I.A., Malik A., Adhami V.M., Asim M., Hafeez B.B., Sarfaraz S., Mukhtar H. (2008). Green tea polyphenol EGCG sensitizes human prostate carcinoma LNCaP cells to TRAIL-mediated apoptosis and synergistically inhibits biomarkers associated with angiogenesis and metastasis. Oncogene.

[59] Shankar S., Chen Q., Siddiqui I., Sarva K., Srivastava R.K. (2007). Sensitization of TRAIL-resistant LNCaP cells by resveratrol (3,4′,5-trihydroxystilbene): Molecular mechanisms and therapeutic potential. J. Mol. Signal..

[60] Yong W.K., Abd Malek S.N. (2015). Xanthohumol induces growth inhibition and apoptosis in Ca Ski human cervical cancer cells. Evid. Based Complement. Altern. Med..

[61] Delmulle L., Vanden Berghe T., Keukeleire D.D., Vandenabeele P. (2008). Treatment of PC3 and DU145 prostate cancer cells by prenylflavonoids from hop (*Humulus lupulus* L.) inducesa caspase-independent form of cell death. Phytother. Res..

[62] Deeb D., Gao X., Jiang H., Arbab A.S., Dulchavsky S.A., Gautam S.C. (2010). Growth inhibitory and apoptosis-inducing effects of xanthohumol, a prenylated chalone present in hops, in human prostate cancer cells. Anticancer Res..

[63] Wei M.C., Zong W.X., Cheng E.H., Lindsten T., Panoutsakopoulou V., Ross A.J., Roth K.A., MacGregor G.R., Thompson C.B., Korsmeyer S.J. (2001). Proapoptotic Bax and Bak:A requisite gateway to mitochondrial dysfunction and death. Science.

[64] Eskes R., Desagher S., Antonsson B., Martinou J.C. (2000). Bid induces the oligomerization and insertion of Bax into the outer mitochondrial membrane. Mol. Cell. Biol..

[65] Catz S.D., Johnson J.L. (2003). Bcl-2 in prostate cancer: A mini review. Apoptosis.

[66] Ray S., Bucur O., Almasan A. (2005). Sensitization of prostate carcinoma cells to Apo2L/TRAIL by a Bcl-2 family protein inhibitor. Apoptosis.

[67] Jiang W., Zhao S., Xu L., Lu Y., Lu Z., Chen C., Ni J., Wan R., Yang L. (2015). The inhibitory effects of xanthohumol, a prenylated chalcone derived from hops, on cell growth and tumorigenesis in human pancreatic cancer. Biomed. Pharmacother..

[68] Kim Y.H., Lee Y.J. (2007). TRAIL apoptosis is enhanced by quercetin through Akt dephosphorylation. J. Cell. Biochem..

[69] Szliszka E., Zydowicz G., Mizgala E., Krol W. (2012). Artepillin c (3,5-diprenyl- 4-hydroxycinnamic acid) sensitizes LNCaP prostate cancer cells to TRAIL-induced apoptosis. Int. J. Oncol..

[70] Szliszka E., Czuba Z.P., Jernas K., Król W. (2008). Dietary flavonoids sensitize HeLa cells to tumor necrosis factor-related apoptosis-inducing ligand (TRAIL). Int. J. Mol. Sci..

[71] Warat M., Szliszka E., Korzonek-Szlacheta I., Król W., Czuba Z.P. (2014). Chrysin, apigenin and acacetin inhibit tumor necrosis factor-related apoptosis-inducing ligand receptor-1 (TRAIL-R1) on activated RAW264.7 macrophages. Int. J. Mol. Sci..

[72] Warat M., Sadowski T., Szliszka E., Król W., Czuba Z.P. (2015). The role of selected flavonols in tumor necrosis factor-related apoptosis-inducing ligand receptor-1 (TRAIL-R1) expression on activated RAW264.7 macrophages. Molecules.

[73] Szliszka E., Kostrzewa-Susłow E., Bronikowska J., Jaworska D., Janeczko T., Czuba Z.P., Krol W. (2012). Synthetic flavanones augment the anticancer effect of tumor necrosis factor-related apoptosis-inducing ligand (TRAIL). Molecules.

[74] Horinaka M., Yoshida T., Shiraishi T., Nakata S., Wakada M., Nakanishi R., Nishino H., Sakai T. (2005). The combination of TRAIL and luteolin enhances apoptosis in human cervical cancer HeLa cells. Biochem. Biophys Res..

